# The challenge of community engagement and informed consent in rural Zambia: an example from a pilot study

**DOI:** 10.1186/s12910-019-0382-x

**Published:** 2019-07-04

**Authors:** Joseph Mumba Zulu, Ingvild Fossgard Sandøy, Karen Marie Moland, Patrick Musonda, Ecloss Munsaka, Astrid Blystad

**Affiliations:** 1 University of Zambia, School of Public Health, P.O. Box 50110, Lusaka, Zambia; 2 Centre for International Health, Department of Global Public Health and Primary Care, University of Bergen, Bergen, Norway; 3 Center for Intervention Science in Maternal and Child Health (CISMAC),Centre for International Health (CIH), University of Bergen, Bergen, Norway; 4 University of Zambia, School of Education, Lusaka, Zambia

**Keywords:** Informed consent, Challenges, Community engagement, Cluster randomized control trial, Zambia

## Abstract

**Background:**

There is a need for empirically based research on social and ethical challenges related to informed consent processes, particularly in studies focusing on adolescent sexual and reproductive health. In a pilot study of a school-based pregnancy prevention intervention in rural Zambia, the majority of the guardians who were asked to consent to their daughters’ participation, refused. In this paper we explore the reasons behind the low participation in the pilot with particular attention to challenges related to the community engagement and informed consent process.

**Methods:**

The pilot was implemented in two schools and examined the acceptability of a package of interventions including economic support to families to keep their girls in school, pocket money for girls, youth club meetings on reproductive health, and community meetings to sensitize the community. Focus group discussions (4) were conducted with girls who participated in the pilot, boys in their class and with parents. Individual semi-structured interviews (11) were conducted with teachers, peer educators and community health workers involved in the coordination of the intervention as well as with religious and traditional leaders. Data were analyzed through thematic analysis.

**Results:**

The findings indicate that inadequate use of recognized community communication channels during the community engagement process and dissemination of information about the pilot resulted in limited understanding of the pilot concept by the community. This surfaced through uncertainty and fear that the intervention may result in loss of control over daughters, worries about why money was provided unconditionally to girls, and suspicion of links to satanism. The sense of insecurity appeared to be exacerbated by low literacy levels, poverty, fear of loss of bride wealth, perceived disregard for local perceptions of social status, and scanty trust in the actors implementing the pilot.

**Conclusions:**

Inadequate use of locally appropriate channels in the dissemination of information created room for interpretation and facilitated development of mistrust, undermining the conditions for community engagement and actual informed consent. A key lesson learnt is the importance of taking seriously the complexity of local values and structures that may impact people’s capability to consent or not consent to a study in an informed manner.

## Background

Informed consent is key to ethical conduct in research. It implies the provision of comprehensive information to potential study participants on the focus of the study, potential risks and benefits of participating in the research, and the securing of voluntary participation in the study without coercion or duress [1–4]. International regulations and guidelines for human subjects in research stress the fundamental ethical principle of seeking voluntary informed consent to participation, and the avoidance of any type of coercion in the processes of recruiting study participants [5]. Meanwhile, the consent process may be influenced or supported by the community engagement activities as there are important interdependencies between the two [3, 4, 6]. According to Tindana et al. [7] the concept of engagement in research refers to “building authentic partnerships, including mutual respect and active, inclusive participation; power sharing and equity; mutual benefit or finding the ‘win–win’ possibility in the collaborative initiative” (p. 1452). Engagement is important as community values, beliefs and norms tend to influence views on risks and benefits of research, thus affecting independent decision making or the consent process [4]. Thus, community engagement may provide insight on how to develop consent processes that are appropriate for the local context [3, 4]. Community engagement activities may also facilitate interpersonal communication which is key to showing respect and making people understand the risks and benefits of the research, as well as appreciating participants’ contributions [6, 8].

Considerable attention has been paid to the dynamics of voluntary consent or assent processes in the recruitment of study participants [2, 3, 9, 10]. The literature reveals that the process of obtaining voluntary consent may be complex [5], and commonly involves factors far beyond the concrete provision of information and the gaining of consent. Recognized challenges in consent/ assent processes include factors such as low literacy levels, language barriers, power inequalities and socio-cultural norms and values that constrain individual autonomy to providing true consent to participation [2, 9, 11]. Practical communication challenges are often linked to the length and format of documents for written informed consent [1, 5, 12, 13].

Conducting sexual and reproductive health (SRH) research with adolescents may raise particular consent and assent challenges, and the literature calls for more research focusing on this group as well as enhanced ethical guidance for research within this topical area due to its substantial sensitivity [14–18]. The challenge may become particularly severe when the study participants are under the consent age, and when the research is conducted in societies with strong cultural and religious norms dictating sexual abstinence, but where youths commonly are sexually engaged. In such instances, guardians of the adolescents may be uncomfortable discussing participation in SRH research due to the strong taboos surrounding the topic [17–20]. Marshall calls for empirical examples of the social and ethical challenges that may emerge in processes of obtaining informed consent in research in this area [2].

Zambia, like other developing countries, is experiencing high pregnancy and early marriage rates among adolescents. Approximately 35% of all 18-year-old girls have given birth and one-third of women aged between 20 and 24 years in 2013/14 were married before the age 18 [21]. Adolescent pregnancies and marriages contribute to a number of SRH challenges, including continued high maternal mortality figures of approximately 400/100, 000 live births [21, 22].

In the preparation for a cluster randomized controlled trial to examine interventions that aim to reduce early pregnancy and early marriage among girls, a pilot study was conducted in Zambia in 2015. The package of interventions included economic support to families to keep their girls in school, pocket money for girls, youth club meetings on reproductive health, and community meetings to sensitize the community. The aim of the pilot was to study the feasibility and acceptability of the planned research procedures and interventions.

A low number of parents or guardians to girls enrolled in grade seven (average age approximately 15 years) consented to their daughters’ participating in the pilot. At school A, 15 out of 26 parents/guardians and their daughters (57%) agreed to participate, while at school B merely six out of 31 parents/guardians and their daughters (19%) agreed to participate in the pilot study. In the present article, we explore the reasons behind the low participation with particular attention to the community engagement and informed consent processes.

## Methods

### Study setting

The pilot study was conducted in two neighbouring schools and in their surrounding communities in a rural district in the Central province of Zambia in September, 2015. The site was purposively selected because of the high numbers of early pregnancies, early marriages and school dropouts among adolescents in the area. The district has a population of 218,328 people.

In Zambia, 31% of those who were aged 20–24 at the time they were interviewed have married before their 18th birthday and 25% of married girls aged 15–19 have an unmet need for family planning. Poverty is also very high as over 70% of the population is living in poverty. Literacy levels among females and males is about 67 and 82% respectively. Zambia has a preamble in its national constitution declaring that it is a Christian Nation, and religious values influence people’s ability to participate in sexual and reproductive health activities [23–26].

### Pilot activities

The present article is based on qualitative interviews and focus group discussions conducted at the end of the pilot study aiming to assess the acceptability of the piloted procedures and interventions. Prior to the pilot study, the District Educational Board Secretary, District Medical Officer, head teachers, and headmen from the selected pilot schools and communities were oriented about the upcoming pilot study. Permission to conduct the study in these areas was obtained. The two schools (A and B) were purposively selected because of their remote location, short distance between them (10 km) and high pregnancies rates among the learners. One teacher from each of the pilot schools, two community health workers (CHWs) and three females (recruited to become peer educators) from the communities around the pilot schools were selected and invited to a three-day training in Lusaka. The headmasters from the two schools coordinated the selection process for the teachers and peer educators, while the officer in-charge of the nearest health facility selected the CHWs to be involved in the pilot. The training focused on sexual and reproductive health education, workshop facilitation and community mobilization.

The following activities were piloted: an orientation meeting for community members / parents and guardians (other relatives taking care of children) of school children about the upcoming intervention study; informing and obtaining of consent from parents/guardians; informing and obtaining of assent from girls enrolled in grade seven; payment of a cash transfers to parents/guardians (ZMW 45) and to girls (ZMW 25); payment of school fees; (two) youth club meetings (one focused on adolescence and puberty, the other on the benefits of education); and finally a community meeting (including role play and discussions about the benefits of education). The pilot was conducted over a period of two weeks.

### Evaluation

Data were collected through focus group discussions (FGDs) with enrolled school children and their parents/guardians, and semi structured interviews (SSI) with involved teachers, community health workers, peer educators, and with community leaders. The community leaders were interviewed to gain a sense of how the community leadership viewed the pilot process. The languages used were Tonga (the local language spoken in the area) and in a few cases English. Data collection was done in September 2015 for a period of one week. FGD and interview guides were used to collect the data. The topics in the guides included experiences with the pilot process, gaps and successes on community engagement, assent and consent processes as well as the recruitment process.

The four FGDs consisted of one group with 10 boys; one group with 10 girls; and two groups with 10 parents in each. The learners were aged between 13 and 15 years. Eleven individual semi-structured interviews were carried out: including with teachers (2); peer educators (3) and community health workers (2) who were involved in implementing the interventions during the pilot, as well as with one religious leader and one traditional leader in charge of the communities (3). Of the eleven SSI, five respondents were female and six were male. Many of the respondents - except for three - had not completed secondary or high school. Parents/guardians and learners who participated in the FGDs were purposively selected from those who had consented to take part in the pilot. We interviewed community leaders who were in-charge of the communities surrounding the schools. Data collection continued until saturation was reached.

The interviews were conducted by four independent research assistants with broad experience in qualitative data collection, three of whom had post graduate training. Two qualitative researchers from the implementing team also conducted a few interviews with key stakeholders. A mix of independent researchers and staff from the project implementation team was seen to be desirable to enhance research credibility. The research team and institution are based in Lusaka and had never conducted research in this site before. Although both the research assistants and the researchers were Zambian, being outsiders in this community (living in the city) and being highly educated might have contributed to the research team struggling to build trust in the community.

The material was audio recorded and transcribed verbatim by the four research assistants. The transcripts were subsequently translated to English. The first author checked the quality of the recordings and transcripts.

### Data analysis

Interviews from the evaluation of the pilot were transcribed and carefully reviewed to generate a codebook of emergent themes [27]. The first author of the paper conducted the initial analysis of the qualitative material from the evaluation of the pilot. A draft set of themes was developed and discussed in the team of authors and subsequently employed to guide the detailed coding process. NVIVO version 7 software was utilized during the coding process (QSR Australia). The codes were grouped into categories related to the diversity of ethical challenges linked to the consent process. The analysis was reviewed by all authors before arriving at the final themes.

### Ethics

The protocol for the pilot study and its evaluation was submitted to the University of Zambia Biomedical Research Ethics Committee for ethical clearance and the Regional Ethical Committee of Western Norway (REK-Vest). The District Educational Board Secretary, District Medical Officer, head teachers and senior headmen gave permission to proceed with the study. The age limit for assent is 17 years and below and the consent age 18 years and above, thus informed consent to participate in the pilot was sought from the adolescents’ parents/guardians and assent was sought from adolescent girls enrolled in grade 7. Before consent was requested from parents/ guardians, a meeting was held where the purpose and content of the pilot was explained. The facilitator of the meeting also explained each section of the consent form in English and received assistance from a local CHW to translate the content into Tonga (the most widely spoken language in the area). Voluntary participation was emphasized during the study recruitment process. Parents were moreover informed that the present research is a pilot study that was to test elements that would potentially be employed in a larger program aiming to reduce early childbearing and school dropout. Girls enrolled in grade 7 at the two selected schools were also provided with information about the purpose and content of the pilot, including the pilot concept itself. The information on the assent form was similarly explained to the girls.

The processes of seeking consent and assent for the evaluation interviews were performed separately at the end of the two-week period through the provision of information about the focus/intention of the evaluation of the pilot, the principles of voluntary participation and the anonymity/confidentiality aspect. Confidentiality was ensured during the data collection and data analysis by using pseudonyms.

## Results

The individual interviews and FGDs carried out at the end of the pilot study indicated that the background for the low participation was a complex web of community engagement challenges such as communication challenges that gave rise to misinterpretations, rumors and fear as well as confusion about the pilot concept, challenges to local ideals and ideas, rumours of satanism and the perceived lack of credibility of the pilot actors. Below we present the key factors that shaped and challenged the consent process in this setting.

### Communication challenges

One of the major gaps in the communication process relates to the non-sharing of initial information about the pilot with all the headmen within the pilot communities. As traditional leaders are important in local communication processes about community activities as socially and culturally denominated gate keepers, the project protocol specified that all the headmen were to be invited to an orientation meeting. This meeting was to take place before the study was introduced to the community to give the headmen an opportunity to learn about the focus of the project and ask questions without the presence of other community members. Nonetheless, only senior headmen attended the first meeting and several of the headmen were not properly informed about the study from their senior headman. As a result, they felt that the research team had not treated them with sufficient respect and became reluctant to actively support the project from the very onset. They were not happy to have received information about orientation meetings about the study through children in school as stated below:

*“The communication process was problematic. Many headmen did not receive information. The procedure is to hold community meetings before starting activities in the community- this was not done” (Community health worker 1, SSI, male, school A).*

*“I can say that they [the parents] will prefer to get information from the headman and the church leaders” (Teacher 1, SSI, male, school A).*

### Confusion about the pilot concept

One of the aspects that shaped the unsatisfactory consent process was an inadequate understanding of the pilot concept among most community members. A headman explained this misunderstanding saying:

“*We have those who did not understand [the pilot]. Others haven’t listened properly”* (Headman 1, SSI, male, school A).

Most parents reported that they did not understand how an intervention program could be conducted solely for 2 weeks. They wondered whether or not the research team would come back to the community as stated by a parent:

“*I was thinking that you will be here for a long time. Why do you want to go back just after a few days? Are you going to come back*?” (Parent 1, SSI, female, school A).

Respondents explained that this confusion was further compounded by the fact that community members had never participated in similar research activities:

“*It is strange, they have found it strange”* (FGD with parents, school A).

Questions about how the community could benefit from participating in the pilot were repeated by several informants. Parents explained in the FGDs that the uncertainty among community members regarding the outcomes of the project affected community participation in the pilot activities as noted below:

“*They are scared because they have not yet seen where it is leading to”* (FGD with parents, school B).

### Challenges to local ideals and ideas

It was reported that community members who prioritized schooling for their children did not have a problem participating in the pilot activities. A youth peer educator explained how prioritization of education affected community participation in the pilot saying:

*“They know their own agenda -so with those who want their children to further their education, they are really in support of this project”* (Youth peer educator 2, SSI, female school B).

The headmen also emphasized that desires to promote education among girls as opposed to early marriage motivated participation in the study:

“*We need to educate the children. A child should not get married”* (Headman 2, SSI, male, school B).

Some of the girls who participated in the youth club meetings reported that they participated because they sympathized with their colleagues who had stopped school due to pregnancy. A youth peer educator explained this sympathy among the girls as follows:

“*The girls (who chose to participate) said they were feeling pity looking at how the girls get pregnant”* (Youth peer educator 1, SSI, female school B).

The dependence upon cattle rearing in the study area, however, brought up the conflicting demands between education of children and the needs for hands for herding. One respondent explained how this dependence on cattle affected participation in the trial:

“*They don’t want their children to learn because what they want is to get something. There are some people who are just singing about cattle herding and getting wealth from the cattle”* (Teacher 1, SSI, male, school A).

Moreover, a series of misunderstandings about the actual content of the intervention emerged, including the contents of the consent form, and its potential implications for the common practice of early marriage in this study setting. Some community members believed that by allowing their children to participate in the pilot and by receiving economic support from the project, they might lose control over their daughters:

“*They think the government is the one paying for their children. They [the parents] will then no longer have the power to bring that child to marriage. If he [the father] gets money from the husband of the child [bride wealth], and he wants the child to get married then maybe the Government will come and arrest that man”* (Teacher 2, SSI, male, school B).

Some of the girls also raised concerns about being arrested:

“*Mmmm… If we stop school before finishing, meaning this money that they give us [will not achieve the intended purpose], they will imprison us”* (Youth peer educator 1, SSI, female, school A).

The practice of early marriage was said to rarely be questioned in the community, and a central reason for the importance of early marriage was its role as a source of income in poverty stricken families. As some community members believed that the intervention might pose a threat to this long-standing custom and central source of income, people became frightened:

“*It was rumored that this study will end the practice of paying of bride price, and that those who receive bride price by marrying off children who are in school would be arrested”* (Headman 2, SSI, male, school B).

The rumors that stiff penalties would be incurred if girls got married before completing school made some parents refuse to sign consent forms. This was explained by a girl saying:

“*Others heard that if you have not finished school, when you get married, your dad will be jailed, that is why others from our school refused”* (FGD with learners, school A).

Learners also mentioned that the refusal by some parents to sign the consent forms was due to parents wanting the girl to stop school and get married as follows:

“*They say , aa … You , … I don’t want you to sign the papers. But they don’t want to tell her directly that they want her to get married”* (FGD with learners, school B).

### Rumours of satanism

With a lack of backing from the headmen, rumours and misunderstandings caught ground. The rumours voiced that the project was linked to satanism and would threaten local values and practices. The fear of associating oneself with something deemed to be satanic particularly frightened and discouraged several community members. The rumours were partly linked to the notion of ‘receiving material goods for free’. People could not understand why a program would be so generous as to provide economic support to girls and their guardians without getting anything tangible in return from the participants, and they did not understand the principle of compensation for participating in interviews or FGDs*.* Some people suspected that the money had ‘charms’ with the potential of bringing misfortune to the family:

“*They say that this money is not genuine money. How can someone give you money without working for it?”* (Teacher 2, SSI, male, school B).

Many perceived receiving money from a program without having done tangible work as a sign of vulnerability, as if they were in a weak position:

*“Mmmm... We told them Why are they helping us? Is it that they have seen that we don’t have money?”*(Community health worker 2, male, school B).

Some community members classified those giving permission for their children to be part of the pilot study as not taking responsibility for the welfare of their children:

“*Other relatives rose up against me saying maybe I have failed to educate the children and now I am thinking of selling them”* (Community health worker 2, male 2, school B). “Many *people thought it was satanism, thinking that maybe they are just playing with our minds”* (Community health worker 2, male 2, school B).

They believed that being part of the pilot would bring bad luck such as death or girls becoming barren:

“*Yes, we have others saying they [the project] are Satanic, or maybe they are blood collectors or whatever, we heard”* (Traditional leader, female, school A).

In some cases, children wished to be part of the pilot but were prevented due to restrictions set by their parents:

**“***Because they fear that it is satanic, their children are interested but the parents are stopping them”* (Youth peer educator 3, female, school B).

Fears about receiving benefits from the pilot such as money were also echoed by the learners. One person explained this fear in the FGD in the following manner:

“*That when you use the money, you will find yourself in a bottle of blood* ”(FGD with learners, school A).

Discussions at both schools with parents and children also confirmed the fear of Satanism as the major barrier to the consent process as outlined in the following statement:

“*Most of them are saying it is satanic, that is the reason why many could not participate”* (FGD with parents, school A).

The community health workers and peer educators who were recruited to champion the pilot activities at the community level were stigmatized and accused of bringing satanism into the community.

“*They said you want to initiate our children into satanism. That is what they said. Others even shouted at us when we reached the villages. They were saying you are surely inviting satanism”* (Youth peer educator 2, female, school B).

Another peer educator confirmed the antagonism in the community:

“*Because they have started thinking ‘how did we get ourselves into it’? We have joined a satanic project. They said ‘No, we are going to stop even to greet you because you joined a satanic project. You have even received bicycles from them [project]”* (Youth peer educator 3, female, school B).

Inability to read, write and comprehend exacerbated the rumors of satanism. A teacher described it as follows:

“*Anything which has to do with signing and writing … - other people they have heard that in satanism you have to sign. He tries to read, but he does not understand anything. The child will say “But why, father, are you not signing?” Then he [the father] will just say - “These are satanic things””* (Teacher 1, male, school A).

In the group discussions, all parents agreed that low literacy levels affected the consent process during the pilot:

“*Lack of information, ignorance, lack of education among parents was a major challenge”* (FDG with parents, school B).

Rumours about satanism did not only affect the recruitment process, but eventually also caused some of the people who had joined the pilot to withdraw from the study:

“*He also has a daughter, but he withdrew. He came to my home, talking about the same [Satanism]. Then he withdrew”* (Religious leader 1, male, school B).

It was reported that similar rumors about satanism had existed in the community before the introduction of the present pilot project. The previous satanism rumors resulted in the rejection of development projects from non-governmental organizations by the community:

“*Airtel would have been here, or the clinic could have been here or secondary schools could have been here. Now - because of people’s fears- that’s why there are no good things”* (Headman 2, SSI, male, school B).

### The perceived lack of credibility of the pilot actors

Limited involvement of several key stakeholders and the traditional leaders in selecting community members who were to be part of the pilot (i.e. youth peer educators and community health workers) moreover affected the acceptability of the pilot. The lack of involvement made community members question both their own and others’ roles in the study.

For the relatively limited numbers who accepted to take part, the awareness of the actors involved in the pilot emerged as decisive. For example, one of the community leaders reported that he was motivated to participate because he was aware that the actors performing the pilot were from an institution that helps people:

“*So you see this doctor, I hurt myself in 2006. He is the one who operated me at the University Teaching Hospital, you see. So it’s just ok - it’s education. If he didn’t get educated, was I going to be there? You see, it’s far, I was at the University Teaching Hospital”* (Headman 3, male, school B).

What is more, for some community members who were able to read and write, the fact that they were able to review and understand the consent form themselves convinced them of the credibility of the research team, and thus were more likely to agree to participate in the pilot. The form had contact details where study participants could forward complaints, which was positively commented upon:

“*Yes … he explained that if you find any problems working with us, there are numbers you can call to report. He left us with those numbers and that’s how we relaxed, because we realized that he was genuine and even gave us numbers to report to. How can a thief tell you” If you think am a thief, report me to the police at this place, unless he is genuine and not a thief? That’s how we realized they were “genuine”* (Religious Leader 1, male, school B).

The letter of support issued on behalf of the Zambian government through the Ministry of General Education (MoGE) was further said to enhance the study’s credibility:

“*The clever ones can accept it because it’s the government doing this. You see. The government is the one directing that you go to this place, otherwise you get lost”* (Headman 3, male, school B).

## Discussion

Several challenges were recorded during this pilot study which significantly affected the consent process of the study. The prime challenge was linked to the initial communication process. The failure to provide information about the study to all headmen and key stakeholders challenged culturally and structurally embedded norms and values regarding hierarchy in this setting. It was perceived as lack of respect and laid grounds for a set of unanswered questions and culturally grounded misunderstandings and misinterpretations. The communication inadequacies moreover enhanced people’s challenges with grasping the purpose and intention of an inherently difficult pilot concept. The belief that the study – which had a focus on reducing early marriage and pregnancy - would threaten the early marriage and bride wealth institution - created obstacles to participation. This in turn led to fear of losing control over daughters, including fear of arrests if they were married off and fear of satanism, partly linked to receiving material goods ‘for free’.

Addressing ethical challenges related to processes of informed consent is vital because if left unaddressed, the obstacles may affect autonomous and voluntary participation in research, including the safety and respect for study participants [5, 7, 9, 13, 28, 29]. We note that in this pilot study the challenges linked to recruitment of participants and the consent process were exacerbated by structural and contextual factors such as poverty and low literacy rates, limiting the extent to which potential participants could freely access information about the study. Low literacy rates may particularly affect participation in contexts where the value of education for many remains a farfetched ideal. Several authors have argued how failure to read or understand consent or assent forms can affect understanding and participation in research [2, 3, 5, 11, 13, 28]. In our case, the lack of reading skills undermined the guardians’ and the girls’ understanding of the pilot.

In order to address the complexity of ethical challenges in consent processes, a fundamental consideration of potential contextual threats to the process, including weak community engagement, is vital. Community engagement is increasingly viewed as a key element of ethical best practice in research [1, 7, 11]. Specifically for the consent process, community engagement is vital as there are interdependencies between consent and community engagement processes in influencing how people view risks and benefits of research thus affecting independent decision making or consenting process [3, 5, 6]. Achieving community participation is, however, not necessarily an easy thing, as we have indicated in this article, and developing a community engagement plan is central [30, 31]. This implies identifying community members who truly represent community needs, including identifying what constitutes a community in a particular context [32]. It moreover implies unpacking what constitutes contextual power relations as these shape community participation dynamics [32]. Community participation in this truly grounded manner can assist in addressing challenges during the research process, not the least by increasing trust between researchers and communities [5, 33] and building “*authentic partnerships, including mutual respect as well as active and inclusive participation”* [7] *.*

The findings of this study further show that despite having the best intentions and plans, sometimes people such leaders and parents may feel disrespected or not well considered if not fully engaged in the design of the research project. Thus, wider and careful engagement processes using locally appropriate channels is vital in ensuring that local concerns are considered when designing and implementing any pilot or intervention study. Lack of such consideration may not only affect the consent process but also affect social relations in the community as those who participate in the research may be stigmatized by the other community members.

Thus, based on the many lessons learnt from the pilot study, we developed a comprehensive community engagement plan for the cluster randomized trial. The plan detailed key stakeholders and the steps to follow in orienting the communities about the trial. The plan for example, included the orientation of the relevant Government Ministries such as Ministry of Chiefs and Traditional Affairs, chiefs, all headmen, religious leaders and other informal leaders before entering the community.

One of the main limitations of the pilot study was inadequate time. Some of the described challenges might have been overcome if the pilot had lasted longer. With regard to the evaluation of the pilot process, the interview guides used for the evaluation did not focus solely on the ethical challenges related to the consent process. Thus, further nuance might have been obtained if we had only focused on and explored more in-depth the recruitment of informants and consent process. We did not explore the disparities between the two schools and we did not interview anyone who declined to participate in the pilot which might have revealed other mechanisms that were at play.

## Conclusion

Participation in the pilot study was very poor due to inadequate use of recognized community communication channels when disseminating information about the pilot. It thus triggered misunderstandings by the community, including fears that participating in the pilot could result in loss of control over daughters and that the project was linked to satanism. Structural factors and local culture surrounding the value of marriage, local notions of social status and perceived trustworthiness or credibility of the actors implementing the pilot moreover influenced the consent process. We stress the importance of taking seriously the complexity of local values and structures when engaging in community communication and engagement processes in research. This is vital especially when sensitive topics such as those involving adolescents’ sexual and reproductive health are to be studied. The process of thoroughly developing a community engagement process that is based on evidence gathered through formative research and strictly followed during the implementation process of the research seems decisive in this context.

## Data Availability

The datasets during and/or analysed during the current study are available from the corresponding author on reasonable request.

## Abbreviations

CHWs: Community health workers
FGDs: Focus group discussions
MoGE: Ministry of General Education
MoH: Ministry of Health

## References

[1] Marsh V, Kamuya D, Rowa Y, Gikonyo C, Molyneux S (2008). Beginning community engagement at a busy biomedical research programme: experiences from the KEMRI CGMRC-Wellcome Trust research Programme, Kilifi, Kenya. Soc Sci Med.

[2] Marshall PA (2006). Informed consent in international Health Research. J. Empir. Res. Hum. Res. Ethics..

[3] Tindana P, de Vries J, Campbell M, Littler K, Seeley J, Marshall P, et al. Community engagement strategies for genomic studies in Africa: a review of the literature. BMC Med Ethics [Internet] 2015;16:24. Available from: http://www.ncbi.nlm.nih.gov/pubmed/25889051%5Cnhttp://www.pubmedcentral.nih.gov/articlerender.fcgi?artid=PMC4407431. 10.1186/s12910-015-0014-z PMC4407431 25889051

[4] Molyneux S, Bull S. Consent and community engagement in diverse research contexts: reviewing and developing research and practice. J Empir Res Hum Res Ethics. 2013. 10.1525/jer.2013.8.4.1 PMC4836561 24169417

[5] Tindana PO, Kass N, Akweongo P (2006). The informed consent process in a rural African setting: a case study of the Kassena-Nankana District of northern Ghana. IRB..

[6] Pratt B, Lwin KM, Zion D, Nosten F, Loff B, Cheah PY. Exploitation and community engagement: can community advisory boards successfully assume a role minimising exploitation in international research? Dev World Bioeth. 2015. 10.1111/dewb.12031 23725206

[7] Tindana PO, Singh JA, Tracy CS, Upshur REG, Daar AS, Singer PA, et al. Grand challenges in global health: community engagement in research in developing countries. PLoS Med [Internet] 2007;4:e273–1455. Available from: 10.1371/journal.pmed.0040273%5Cnhttp://proxy.lib.sfu.ca/login?url= https://www.ncbi.nlm.nih.gov/pubmed/17850178 10.1371/journal.pmed.0040273 PMC1989740 17850178

[8] Njue M, Molyneux S, Kombe F, Mwalukore S, Kamuya D, Marsh V. Benefits in cash or in kind? A community consultation on types of benefits in health research on the Kenyan coast. PLoS One. 2015. 10.1371/journal.pone.0127842 PMC4444261 26010783

[9] Kass NE, Maman S, Atkinson J (2005). Motivations, understanding, and voluntariness in international randomized trials. IRB Ethics Hum Res.

[10] Woodsong Cynthia, Karim Quarraisha Abdool (2005). A Model Designed to Enhance Informed Consent: Experiences From the HIV Prevention Trials Network. American Journal of Public Health.

[11] Marsh VM, Kamuya DM, Mlamba AM, Williams TN, Molyneux SS (2010). Experiences with community engagement and informed consent in a genetic cohort study of severe childhood diseases in Kenya. BMC Med. Ethics [Internet]..

[12] Zulu Joseph Mumba, Lisulo Mpala Mwanza, Besa Ellen, Kaonga Patrick, Chisenga Caroline C., Chomba Mumba, Simuyandi Michelo, Banda Rosemary, Kelly Paul (2014). Improving Validity of Informed Consent for Biomedical Research in Zambia Using a Laboratory Exposure Intervention. PLoS ONE.

[13] S.R. H, M.C. S-M, P. H, Y. N. Framing and personalizing informed consent to prevent negative expectations: an experimental pilot study. Heal Psychol [Internet] 2015;34:1033–1037. Available from: http://www.embase.com/search/results?subaction=viewrecord&from=export&id=L602331767%5Cnhttp://dx.doi.org/10.1037/hea0000217%5Cnhttp://sfx.library.uu.nl/utrecht?sid=EMBASE&issn=19307810&id=doi:10.1037/hea0000217&atitle=Framing+and+Personalizing+Informed+Co 10.1037/hea0000217 25689300

[14] Ringheim K. Ethical issues in postabortion care research involving vulnerable subjects. In: Huntington D P-PNJ, editor. Postabortion Care Lessons from Oper. Res. New York: The Population Council; 1999. p. 178–197.

[15] Gipson JD, Becker D, Mishtal JZ, Norris AH (2011). Conducting collaborative abortion research in international settings. Womens Health Issues.

[16] Council for International Organizations of Medical Sciences & World Health Organization. International ethical guidelines for biomedical research involving human subjects. [Internet]. Bull. Med. Ethics 2002 p. 17–23. Available from: https://cioms.ch/wp-content/uploads/2017/01/WEB-CIOMS-EthicalGuidelines.pdf. 14983848

[17] Hess R (2006). Postabortion research: methodological and ethical issues. Qual Health Res.

[18] H S, Andersson C, Janzon LSN (1998). Selection bias in a study on how women experienced induced abortion. Eur J Obs Gynecol Reprod Biol.

[19] Adler NE, David HP, Major BN, Roth SH, Russo NF, Wyatt GE (1990). Psychological responses after abortion. Science..

[20] Helitzer-Allen D, Makhambera M, Wangel AM (1994). Obtaining sensitive information: the need for more than focus groups. Reprod Health Matters.

[21] Central Statistical Office (CSO) [Zambia], Ministry of Health (MOH) [Zambia] and II. Zambia demographic and health survey 2013–14. Lusaka; 2014.

[22] Muzira T, Njelesani M ZJ. The condition of young people UN Zambia signature issues series - # 2. 2013.

[23] Haynes N. (2015). "Zambia Shall be Saved!": Prosperity Gospel Politics in a Self-Proclaimed Christian Nation. Nova Religio: The Journal of Alternative and Emergent Religions.

[24] Mwansa A. Law, religion and human rights in Zambia: the past, present and the practice. African Hum. rights law J. [Internet]. 2008;8:546–67. Available from: https://search.proquest.com/docview/37207276?accountid=13042%0Ahttp://oxfordsfx.hosted.exlibrisgroup.com/oxford?url_ver=Z39.88-2004&rft_val_fmt=info:ofi/fmt:kev:mtx:journal&genre=article&sid=ProQ:ProQ%3Aibss&atitle=Law%2C+religion+and+human+rights+in+Zamb.

[25] Cheyeka A, Hinfelaar M, Udelhoven B (2014). The changing face of Zambia’s Christianity and its implications for the public sphere: a case study of Bauleni township, Lusaka. J South Afr Stud.

[26] Zulu JM, Ali J, Hallez K, Kass NE, Michelo C, Hyder AA. Ethical challenges in research on post-abortion care with adolescents: experiences of researchers in Zambia. Global Bioethics. 2018:1–16. 10.1080/11287462.2018.1528657 PMC7733980 33343186

[27] Braun V, Clarke V, Braun V, Clarke V (2006). Using thematic analysis in psychology., 3:2 (2006), 77-101. Qual Res Psychol.

[28] Edmund Howe JDM (2008). Beyond informed consent: the Ethics of informing, anticipating, and warning. Psychiatry (Edgmont).

[29] Kass NE (2001). An Ethics framework for public health. Am J Public Health.

[30] Rifkin SB, Muller F, Bichmann W (1988). Primary health care: on measuring participation. Soc Sci Med.

[31] Rifkin SB (2009). Lessons from community participation in health programmes: a review of the post Alma-Ata experience. Int Health.

[32] Rifkin Susan B. (1996). Paradigms lost: Toward a new understanding of community participation in health programmes. Acta Tropica.

[33] Childress JF, Faden RR, Gaare RD, Gostin LO, Kahn J, Bonnie RJ (2002). Public health ethics: mapping the terrain. J Law Med Ethics.

